# A qualitative evaluation of a simplified cardiovascular management program in Tibet, China

**DOI:** 10.1186/s12992-018-0342-0

**Published:** 2018-03-01

**Authors:** Maoyi Tian, Xuejun Yin, Danzeng Dunzhu, Zhong Liu, Cong Li, Hao Sun, Ci Song, Laba Sangzhu, Anushka Patel, Julie Redfern, Lijing L. Yan

**Affiliations:** 1 The George Institute for Global Health at Peking University Health Science Center, Beijing, China; 2 The George Institute for Global Health, University of New South Wales, Sydney, Australia; 3 Sydney Medical School, University of Sydney, Sydney, 2006 NSW Australia; 4 Beijing University of Chinese Medicine, Beijing, China; 5 Tibet University, Lhasa, China; 6 Peking University Health Science Center, Beijing, China; 7 China Medical University, Shenyang, China; 8 Global Health Research Center, Duke Kunshan University, 8 Duke Avenue, Kunshan, Jiangsu 215347 China

**Keywords:** Qualitative study, Cardiovascular diseases, Village doctor, Tibet, Rural health

## Abstract

**Background:**

The simplified cardiovascular management (SimCard Study) program was a cluster randomized controlled trial conducted in Tibet, China to evaluate a multifaceted intervention consisting of appropriate medication prescriptions and lifestyle recommendations delivered by village doctors. The intervention was effective in improving the management of cardiovascular diseases in resource-limited settings. The aim of this qualitative study was to examine stakeholder feedback and to inform future research and scaling up.

**Method:**

A total of 28 face-to-face individual interviews were conducted. The interviews were conducted in 6 out of 14 intervention villages by 2 interviewers who speak the local language. Participants included 18 community members at high risk of CVD, 6 village doctors, 2 local project coordinators, and 2 county officials. Interview guides were used to facilitate the interview covering the focus of perceived usefulness and content of the intervention, fidelity to the intervention, and potential scalability of the intervention. Qualitative interviews were coded using thematic analysis.

**Results:**

The average age of the participants was 41 years and 70% were female. Our findings showed that the intervention was delivered according to the protocol and was described as a useful program for CVD management by both high-risk individuals and village doctors. However, lack of knowledge among high-risk individuals, insufficient availability of healthcare providers, inadequate financial incentive, and incomplete infrastructure such as difficulty in transportation and cell phone signal were identified as the main barriers to successful implementation and scale-up.

**Conclusion:**

The intervention was implemented in line with the protocol and provided substantial benefits for relevant community members and health professionals. However, multiple health system barriers need to be addressed for successful scale-up in rural China.

**Trial registration:**

Unique identifier: NCT01503814. Registered 11 December 2011.

## Background

Globally, 17.5 million deaths were attributed to cardiovascular disease (CVD) in 2012 [1]. Over 75% of those deaths took place in low- and middle income-countries [1]. In China, CVD was responsible for more than 40% of all deaths in both urban and rural populations in 2015 [2]. Modifiable risk factors for CVD include high blood pressure (BP), unhealthy diet, physical inactivity, and tobacco use [3]. This growing disease burden and ineffective management of modifiable risk factors pose a significant challenge for the Chinese healthcare system [4]. Resource-constrained areas with ill equipped primary healthcare services are of particular concern [4]. Tibet Autonomous Region (Tibet) lies in south-western China. Due to the rough terrain and high altitude, Tibet remains one of the most remote and undeveloped areas in China. This is not only reflected in the social economics but in healthcare [5]. Recent studies conducted in Tibet found more than 50% of herdsmen aged 40 years and above had high BP [6].

Our group recently developed, implemented and evaluated a SIMplified CARDiovascular (SimCard) Management Program in Tibet, China and Haryana, India [7, 8]. The overall study results showed that, at 12-month follow-up, there was a significant 26% increase in the proportion of intervention participants being treated with low dose diuretics and aspirin and a significant 2.7 mmHg reduction on the systolic blood pressure (SBP), compared to control group participants [8]. With stratification by country, China and India had similar results on the primary outcome (the proportion of antihypertensive medication use). However, China showed a larger significant reduction on SBP (4 mmHg). It was concluded that the simplified cardiovascular management program effectively improved the quality of clinical care at the primary care level in resource-limited settings in both countries.

Although the SimCard Study adopted a simplified intervention as compared to clinical guidelines, it was a complex trial with multiple components. The published quantitative outcomes [8] cannot ascertain how the intervention was implemented or the extent to which the intervention might be scalable. The use of a qualitative approach alongside randomized controlled trials (RCTs) is becoming more common and has the potential to better understand the effects of components of complex interventions, assess intervention fidelity, and evaluate the acceptability and scalability of the intervention [9, 10]. To achieve these aims, we performed a qualitative evaluation in parallel with this intervention, as recommended for evaluation of trials with complex interventions [11–13]. This paper describes the findings for China.

## Methods

### Design

The qualitative descriptive approach was used to inform this evaluaiton. Qualitative interviews were conducted after the final evaluation within the SimCard Study. The SimCard study protocol was published and the trial is registered (NCT01503814) [7]. The study, including the qualitative interviews, was approved by the Institutional Review Boards of the Peking University Health Science Center and Duke University. All participants involved in the qualitative study provided informed consent.

### SimCard main trial design

SimCard was a single blinded cluster RCT that aimed to evaluate a guideline-based cardiovascular management program delivered by the community health workers (CHWs) [8]. The CHWs in rural China are known as “village doctors”, who are primary care providers in rural settings who have received basic medical and pharmaceutical training and can prescribe medications. For the SimCard Study, 27 villages (involving 1036 individuals at high risk of CVD) from 2 counties in Tibet, China (Gongbujangda and Linzhou) were recruited. Those high-risk individuals who were residents of the intervention villages were advised to participate in lifestyle modification (smoking cessation and salt reduction) and to receive evidence-based prescription of two medications (low-dose diuretics and aspirin). The intervention lasted for one year, and was delivered by the trained village doctors monthly with the assistance of a smartphone-based electronic decision support system (EDSS). Before the start of the intervention, all village doctors in the intervention villages received a one-day systematic training, with refresher training provided every 3–4 months. Performance feedback to and performance-based incentives for the village doctors were also part of the intervention.

### Setting

Interviews were conducted in 6 intervention villages: 3 villages in Gongbujiangda County and 3 villages in Linzhou County. Villages in each county were selected purposively based on the maximum variability in their overall performance during the intervention. The overall performance was assessed based on a mix of objective and subjective measurements. The objective measurements were key performance indicators including the percentage of high-risk individuals receiving regular follow-up, lifestyle advice, and medications. The subjective assessments were provided by the study local coordinators in each county, based on their general impression of performance.

### Participants

A total of 28 subjects participated in the qualitative interview. These participants represent different stakeholders of the SimCard intervention. First, community members (3 per village) in SimCard Study who were at high risk of CVD were recruited. High-risk community members (hereafter named HR) were selected based upon their level of participation in the study. Level of the participation was determined by the percentage of the total follow-up visits attended during the intervention (High: ≥70%; Moderate: > 30% and < 70%; Low: ≤30%). For each participating village, a list of all high-risk individuals and their level of participation in the study was generated. In this list, high-risk individuals were listed in random order by the three participation levels and one individual in each category level was selected based upon their position on the list, beginning from the top of the list. If the selected individual was not available on the day of the evaluation, then the next individual listed in the same participation category was selected. Table 1 summarises the intervention outcomes of the high-risk participants at baseline and follow-up. Second, six village doctors (hereafter named VD, each village has only 1 such individual) who implemented the intervention for the main trial, were interviewed. Third, two project coordinators (hereafter named PC, 1 per county) who were the township physician in Gongbujiangda County and the Center for Disease Control officer in Linzhou County were interviewed. Finally, two county officials (hereafter named CO, 1 per county who were the President of the County Hospital in Gongbujiangda County and the Director the County Health Bureau in Linzhou County) were interviewed.

**Table 1:** SimCard Study outcomes for the community members at high-risk of CVD from baseline to follow-up

Outcomes (Mean ± SD or %)	Baseline	12-month follow-up
Use of low dose diuretics in the past month, %	0	67
Use of aspirin in the past month, %	0.7	73
Systolic blood pressure, mmHg	149.2 ± 25.4	136.5 ± 15.9
Diastolic blood pressure, mmHg	95.6 ± 14.9	91.3 ± 9.4
Awareness of harm of high salt diet, %	60	100
Current smoker, %	40	30
Receiving monthly follow-up, %	33	80
Hospitalization during the past year, %	13	20

### Data collection

All interviews were conducted after the post-intervention survey of the SimCard study. As participants were located in geographically dispersed locations, the use of face-to-face interviews were adopted as the most feasible method to collect data. The interviewers were conducted by two experienced researchers from Tibet University who were fluent in the local language. All interviewers were blinded to the study outcomes and had not played any role in the SimCard trial. Interviews followed a pre-prepared interview guide containing a mixture of close- and open-ended questions. The initial interview guide was developed after discussion among the multi-disciplinary research team. The guide was further modified based on the feedback provided by the interviewers to accommodate the local culture and language before the formal interviews. Separate interview guides were used for different group of participants. Table 2 shows the interview key themes for each group of participants. All interviews were conducted in a quiet and private room to allow participants to share their views confidentially without being influenced by others. All interviews were audio-recorded and transcribed verbatim by the interviewers in Tibetan local language. Transcripts were then translated into Mandarin Chinese by the interviewers. The translated transcripts were verified by an independent researcher who was fluent in both Tibetan and Chinese.

**Table 2:** Interview contents for each group of the interview participants

Participant Category	Interview guide focus
County officials and county-level project coordinators (4 participants)	Demographic information Perceived usefulness Scalability
Village doctors (6 participants)	Demographic information Perceived usefulness Fidelity to the intervention Intervention content Scalability
Representative high-risk individuals (18 participants)	Demographic information Perceived usefulness Fidelity to the intervention Intervention content

### Data analysis

Demographic information and categorical results are summarized as means and proportions. Qualitative interviews were coded using thematic analysis to identify the common threads across the interviews [14]. The thematic method consists of five steps: (1) Gain familiarity with the entire data by listening to the recordings and reading the transcripts; (2) Generate codes by capturing the important features of the data to guide the analysis. Two researchers (MT, XY) independently manually coded each transcript line by line, using highlighters; (3) Search for themes by identifying similarities across the data set; (4) Review, revise, and refine the candidate themes. (5) Define and name the themes by identifying the key components of each theme and constructing an informative name.

All discrepancies in coding between the two researchers were discussed with disagreements resolved by consensus. Quotations used in this paper were translated to English and then back translated into Mandarin Chinese. The back translations were compared with the original Mandarin Chinese texts to confirm accuracy.

## Results

A total of 28 participants were interviewed. Their mean age was 48 (± 15) years and the majority were female (75%). The interviews were conducted between July 2013 and August 2013, and lasted from 0.5 h to 1.5 h. The demographic information was summarized in Table 3 for each group of participants. Table 4 summaries the key themes identified and major findings under each theme.

**Table 3:** Demographic information of all interview participants

Characteristics (Mean ± SD or %)	High-risk individuals (*n* = 18)	Village doctors (*n* = 6)	County officials and project coordinators (*n* = 4)
Age (years)	56 ± 10	31 ± 11	36 ± 8
Female (%)	77.8	83.3	50.0
Highest education level			
Illiterate	66.7	0	0
Primary school	27.7	16.7	0
Secondary school	5.6	66.6	0
University and above	0	16.7	100
Body mass index, kg/m^2^	22.7 ± 3.8	–	–
Waist circumference, cm	94.1 ± 7.2	–	–
Disease history, %			
Coronary heart disease	80	–	–
Stroke	20	–	–
Diabetes mellitus	0	–	–

**Table 4:** Key themes identified and major findings

Key themes	Major findings
Perceived usefulness of the risk management program	• High-risk community members were satisfied with the intervention. • Most villages doctors maintained a positive attitude on the program.
Content of risk management program	• EDSS was a promising tool for CVD management. • EDSS needed to be designed in local language. • Mobile or wireless internet signals needed to be improved. • The use of western medications was a concern among the high-risk community members.
Fidelity to the risk management program	• Lack of knowledge, and traditional cultural belief were the main barriers to medication adherence. • Village doctors showed a high level of fidelity to the intervention.
Barriers and facilitators to implementation	• Lack of trained personnel, large existing workload, financial incentive, and transportation were identified as major challenges for implementation.

### Perceived usefulness of the risk management program

All of those at high risk of CVD interviewed were positive about the program. Most said the program should be continued and was useful with example quotations provided below.

*“The treatment provided was very helpful to me, my blood pressure is much lower than before.”* (High-risk individual, HR07)

*“I hope this program will continue to be implemented.”* (High-risk individual, HR12)

*“This program provided me free medications. The village doctor visited me at my home every month. All those brought me a lot of convenience.”* (High-risk individual, HR05)

Similarly, all village doctors reported a positive attitude on the program with illustrative quotes provided below.

*“This was a beneficial program which provides free medications. This helped the patients to relieve from their illness. This program not only won the praise from the patients, but also gave me the opportunity to grow myself. I feel proud to serve the villagers in my hometown.”* (Village doctor, VD03)

*“This program helped me to improve my professional knowledge. I hope it can be continued.”* (Village doctor, VD05)

### Content of risk management program

In terms of the intervention content, more community members at high-risk of CVD believed the Tibetan traditional medicine (39%) was superior to western medicine (28%), such as the low dose diuretics provided in the study. Most of the high-risk community members (80%) reported the health education promotional materials were useful.

*“I think it (the poster) was pretty good. I can read the messages from the pictures even if I am illiterate.”* (High-risk individual, HR01)

*“It (the poster) was posted on the wall of the corridor, which provided a very good reminder for the whole family.”* (High-risk individual, HR14)

All village doctors reported to use the smartphone-based EDSS during their regular follow up. An average score of 4 was received on a scale of 1-5 from the village doctors (1 means not helpful at all, burdensome; 5 means very helpful, facilitated greatly). However, the village doctors experienced obstacles while using the smartphone mainly reflecting on the mobile signal and the language of the user interface:

*“It is better to be translated because my Mandarin Chinese level is very low.”* (Village doctor, VD01)

*“There were always problems when uploading the data due to the bad signal.”* (Village doctor, VD04)

*“I can’t upload the data on time as required because of the poor connection.”* (Village doctor, VD05)

### Fidelity to the risk management program

While there was a significant increase on the use of the medications, there was still resistance to use of western medicines on the part of the community members at high CVD risk. The major reasons were due to culture beliefs and concerns about side effects:

*“I thought Tibetan herb is more effective for chronic conditions.”* (High-risk individual, HR17)

*“I stopped to take the medicines when I heard from the other villagers that the medicines have severe side effects.”* (High-risk individual, HR11)

*“I only took the medicines when I have very severe symptoms.”* (High-risk individual, HR09)

“*I only took Tibetan medicines when I am sick.*” (High-risk individual, HR13)

All community members reported attempts to reduce salt intake through food and drink preparation and consumption.

“*I am aware that the maximum daily intake of salt is up to 6 grams.*” (High-risk individual, HR12)

*“I reduced a lot of salt when I cook and drink the yak-butter tea.”* (High-risk individual, HR07)

*“I used to drink a lot of yak-butter tea every day, but now I change to drink water instead.”* (High-risk individual, HR03)

However, community members found it hard to adhere to the salt reduction because:

*“The food tastes very bad with less salt.”* (High-risk individual, HR09)

*“There is no flavour (when adding less salt).”* (High-risk individual, HR08)

All village doctors reported provided the lifestyle recommendations to high-risk individuals as outlined in the study intervention protocol:

*“I advised the patients not exceeding 6 grams of salt per day. If they don’t know how much is 6 grams, I will suggest them not exceeding the amount of a cap for a glass beer bottle.”* (Village doctor, VD04)

*“I asked the patients to hang the posters on the wall of their kitchen for better reminding.”* (Village doctor, VD05)

All village doctors indicated adherence to the medication management plan, and provided information to the patients in relation to the recommended dosage and potential side effect.

*“I addressed the dosage to the patients when I distribute the medication. Aspirin shall be taken once a day, three pills each time. Diuretics, once a day, one pill each time.”* (Village doctor, VD04)

*“I also informed the patients about the potential side effects of the medications.”* (Village doctor, VD05)

### Barriers and facilitators to implementation

All village doctors suggested the program was feasible to be scaled-up in other areas in Tibet. They indicated two major barriers need to be considered for scalability:

*“Transportation will be the main barrier if the program is extended to other pastoral areas.”* (Village doctor, VD05)

*“The program will be hard to be implemented in other areas if without local government (administrative) support.”* (Village doctor, VD06)

While all county project administrators provided a positive comment on the program, there indicated many likely barriers for widespread implementation:

*“The education level of current village doctors is very low, and we don't have enough workforce. The central government requires one village doctor serving a radius of 5 Km. We are obviously falling behind.”* (County official, CO01)

*"If this program will be scaled up to the whole Tibet region, the major barrier will be the transportation, particularly for those in very remote areas."* (Project coordinator, PC02)

*"The current incentive scheme will not motivate village doctors at all."* (County official, CO02)

## Discussion

The quantitative evaluation of the SimCard Study showed that the multifaceted intervention model significantly improved the quality of care for CVD management at the primary care level in resource-limited settings [8]. However, the improved outcomes were only observed in the pharmaceutical component of the program, not with respect to lifestyle. This qualitative study, conducted in parallel, aimed to capture the opinions of a range of participants involved in this program, explore the reasons for the success and failure, and provide recommendations for future intervention programs in a similar, but large-scale scale-up setting. The findings suggest that the improvements observed were consistent with reported implementation according to the protocol; collectively this indicates fidelity was likely to be good. The intervention was described as meaningful and worth scaling up in resource-limited settings. However, our qualitative findings also highlighted the obstacles to scaled-up implementation and areas for improvement.

The intervention model was adapted from the previous studies in China [15], and was designed with feasibility and suitability to local context in mind. All participants in the intervention indicated a positive attitude to the program and were satisfied with the content. These findings were consistent with the effectiveness of the intervention demonstrated by the quantitative evaluation on changing the healthcare provider and patient behaviour, and improvements in the clinical outcome of SBP [8].

The use of EDSS was the first of its kind to be implemented in this setting for the management of CVD. Although the village doctors provided very promising feedback on the acceptability of this innovative technology, the findings from this qualitative evaluation revealed barriers to the use of EDSS and provide recommendations for the future development. Software design in local language and wider, more reliable coverage of the mobile or wireless internet networks will be the main technology requirements for scale-up.

While the quantitative fidelity indicators were only monitored during the implementation, including if the village doctors provided the monthly follow-up, if the health educational materials were distributed, and if the EDSS was used during the follow-up, the qualitative study will provide some supportive information related to the fidelity measurement. Although based on self-report, the qualitative component supports a high level of fidelity to the intervention program. In China, the village doctors are tasked by the government to provide basic primary health care as CHWs. They typically lacked training and capacity to treat chronic diseases. With the systematic and periodic refresher training provided, as well as the assistance of the EDSS, both quantitative and qualitative results indicate that the village doctors’ clinical competence improved and adherence to the intervention program was good. From the patients’ perspective, while there was a willingness to modify behaviour, lack of knowledge and alternate traditional cultural beliefs were the main barriers to medication and lifestyle adherence. A component of intensive education to the patients is suggested to improve the patients’ adherence in future research.

Workforce shortages, lack of financial incentives, and transportation issues were identified as other major challenges for future scale-up. In Tibet, on average, only one village doctor is available per village, who serves a mean population of 1000 and is responsible for all basic healthcare services, including infectious disease surveillance, vaccination, maternal and child health maintenance, as well as the prevention and management of non-communicable diseases. The annual salary that the village doctor receives from the government is about US$500, which is far less than the China Gross Domestic Product Per Capita. Village doctors’ current focus is on disease treatment rather than preventive care, and curative care consumes most of their salaried time. Although the village doctors receive subsidies from the government for their work on preventive care, the amount remains small. The limited amount of incentives ultimately influences the motivation of the village doctors, particularly on the preventive care. In Tibet, China, the population is widely distributed across high mountain areas. Lack of reliable and accessible transportation serves as another barrier for the village doctors to deliver healthcare. Transportation may not be an issue that the health sector can deal with by itself, but will be a key infrastructure for the central or local government to consider.

The qualitative findings in the SimCard trial were consistent with findings from other studies. For example, a study utilising data from the 2011 China Primary Care Workforce Survey assessed key performance motivating factors for community-based primary care providers [16, 17]. The study found the most important motivating factors were professional training, living environment, work benefits, working conditions and income [16]. The study also indicated that there was a greater need for improvement in rural than urban settings [16]. Another study used the same database to examine the factors associated with the job satisfaction among Chinese rural village doctors [17]. The results demonstrated that low income and high workload were the two major contributing factors towards job dissatisfaction [17]. While the results of the SimCard trial suggested a positive attitude of the primary care providers towards CVD management, new comprehensive training and incentive mechanisms for primary care providers, particularly the rural village doctors, is required for a sustainable approach to improving CVD prevention and management.

There were several strengths and limitations of this study. The qualitative design allowed the analysis of the barriers to each component of the complex intervention, which was difficult to capture quantitatively. The qualitative interviews involved a range of stakeholders, to generate a comprehensive understanding of the content and perceived usefulness of the intervention. However, due to language barriers, despite efforts to back-translate the transcript to ensure the content captured the intended meaning, the study remained descriptive and was not conducive to further in-depth exploration.

## Conclusion

The SimCard study provided evidence in support of an effective intervention model for CVD management in resource-limited settings, however despite its acceptability, multiple barriers to scale up were identified. Addressing these barriers in the current health system and for other similar interventions will be critical for the improvement of CVD management in rural China.

## Abbreviations

BP: Blood pressure
CHW: Community health worker
CO: County official
CVD: Cardiovascular disease
EDSS: Electronic decision support system
HR: High-risk community member
PC: Project coordinator
RCT: Randomized controlled trial
SBP: Systolic blood pressure
SimCard: Simplified cardiovascular management program
Tibet: Tibet autonomous region
VD: Village doctor
